# Are dark-eyed dogs favoured by humans? Domestication as a potential driver of iris colour difference between dogs and wolves

**DOI:** 10.1098/rsos.230854

**Published:** 2023-12-20

**Authors:** Akitsugu Konno, Hitomi Aoki, Emiri Suzuki, Seiya Furuta, Sayoko Ueda

**Affiliations:** ^1^ Department of Animal Sciences, Teikyo University of Science, Yamanashi, Japan; ^2^ The Mt. Fuji Institute for Nature and Biology, Showa University, Yamanashi, Japan

**Keywords:** dogs, domestication, evolution, eye, iris colour, wolves

## Abstract

Comparative studies have shown that the eye morphology of primates has been shaped by a variety of selection pressures (e.g. communication, environmental factors). To comprehensively elucidate the complex links between ocular morphology and its evolutionary drive, attention should be paid to other phylogenetic groups. Here, we address a new question regarding the evolution of eye colour patterns in the oldest domesticated animal, namely, the domestic dog (*Canis familiaris*). In this study, we conducted an image analysis of dogs and their closest relatives, grey wolves (*Canis lupus*), to compare the colours of their irises, with the aim of assessing whether eye colours of dogs affect how humans perceived dogs. We found that the irises of dogs were significantly darker than those of wolves. We also found that facial images of dark-eyed dogs were perceived as more friendly and immature, potentially eliciting caregiving responses from humans. Our findings are consistent with our expectation that humans favour dark-eyed dogs over light-eyed ones and provide an updated hypothesis that dogs with dark eyes may have evolved by acquiring a facial trait that sends a non-threatening gaze signal to humans.

## 1. Introduction

The eyes serve as both an input and output device for visual information. The pioneering research by Kobayashi and Kohshima has characterized the human eye as being different from other primate eyes; specifically, it has white sclera that is more widely exposed and horizontally elongated than that in other primates, possibly enhancing the visibility of gaze directions [1,2]. Recent comparative image analyses of humans and great apes provide updated evidence that humans have a uniformly white sclera, which offers clear visibility of both the eye outline and iris [3,4]. Moreover, a comparative behavioural study suggests that both humans and chimpanzees more easily discriminate the directions of eye-gaze from human eye image than chimpanzee eye image [4]. These findings support the idea that salient eye features with white sclera and dark iris in humans enhance communicative interactions with eye-gaze signals (especially in the context of gaze or glancing direction), contributing to the maintenance of cooperative relationships among group members [5].

Conversely, questions have been raised regarding the association between the uniqueness of eye features in humans and their communicative function. Comparative studies of eye morphology have shown that other great apes have similar colour (contrast) differences between the iris and sclera and scleral exposure to those of humans, with highly individual differences in scleral depigmentation [6–9]. Moreover, the findings by Caspar *et al*. [6] do not show an association between the eye colour pattern and social cognition in ape species. This indicates that eye coloration in apes may have been catalysed by different evolutionary mechanisms (e.g. genetic drift and sexual selection). Perea-García *et al*. [10] demonstrated that the variation of eye shape and colour, across 77 primates, can be explained by their habitat (i.e. terrestriality, latitude), suggesting the higher importance of ecological factors on the eye morphology of non-human primates rather than communicative ones. In line with these results, Perea-García *et al*. [11] identified five eye morphology traits (i.e. dark corneal limbus, temporal wedge, white corneal limbus, iris and pupil, and local variation in scleral pigmentation) that have not received attention and proposed that these traits may have social communicative functions in some species (see the seventh paragraph of the introduction in detail). Their review also argues that other potential adaptive values of eye morphology including photoprotective and photoregulatory functions should be comprehensively investigated.

A comparative approach facilitates the comprehension of the complex links between ocular morphology and its evolutionary drivers. While some previous studies have focused on eye colour and its functions in avians, amphibians and fishes (e.g. [12–14]), only one study has been conducted on the eye morphology of non-primate lineages in mammals. Ueda *et al*. [15] focused on wild canid species and found that the eye and facial colour patterns of canids are associated with their social structure and gaze communication. Their image analysis demonstrated that most wolf-like canids (i.e. grey wolves, red wolves, coyotes, golden jackals, Ethiopian wolves, dholes, black-backed and side-striped jackals) have a distinct eye colour type with light-coloured irises and tend to have standard lifestyles living in family groups and engage in group hunting. These findings imply that salient eye colour patterns in wolf-like canids may be correlated with conspecific communicative functions. However, this notion is speculative rather than empirical because the experimental evidence showing that light-eyed canids have more specialized social communicative abilities than dark-eyed canids is critically lacking.

**Figure 1.**
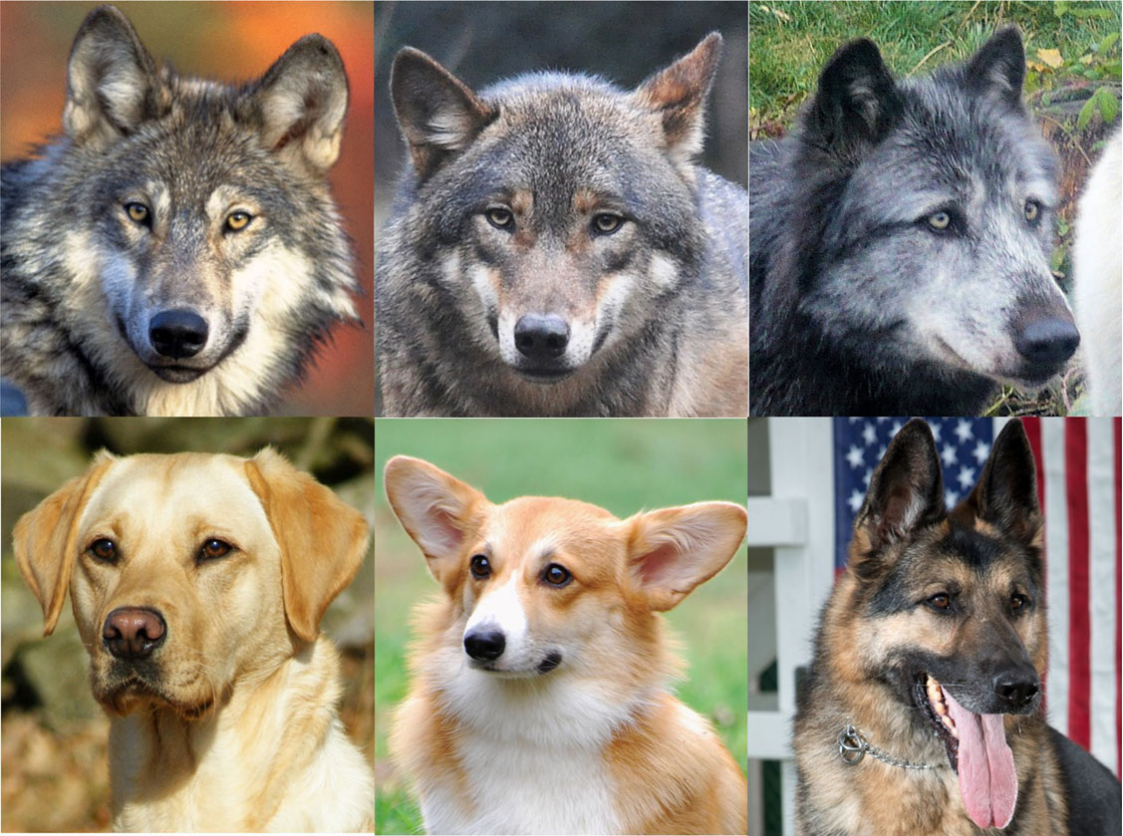
Examples of facial (eye) morphology in wolves and dogs. The eyes (iris colour) of domestic dogs appear to be darker than those of wolves.

Here, we address a new question regarding the evolution of eye colour patterns in the oldest domesticated animal, the domestic dog (*Canis familiaris*). Dogs were domesticated from grey wolves at least 15 000–50 000 years ago, through both natural and artificial selection [16,17]. The eye colour of domestic dogs appears to be darker than that of grey wolves (figure 1). The eye colour of both dogs and wolves is mainly determined by the iris colour because most of the sclera is not exposed externally and their pupils are commonly dark. The iris colour of wolves is typically classified as yellow [18], whereas that of dogs can be described as dark brown or black. A darker iris colour is recommended in breeding programmes for most pure-bred dogs. We summarized the description of eye colour in the breed standard (i.e. a description of the characteristics of a hypothetical or ideal example of a breed) and found that dark-coloured eyes are recommended for 77 of the 82 primary breeds (93.90%) in the American Kennel Club and for 76 of the 82 breeds in the Kennel Club (92.68%) (see electronic supplementary material and discussion). These qualitative data suggest that dogs have evolved darker eye (iris) colours than wolves. However, there are no comparative studies on the eye colour of dogs and wolves, especially in the context of image analysis.

Next, we address the further issue that the domestication process may have favoured dark-eyed dogs over light-eyed dogs and explore how eye colour differences (darker iris versus lighter iris) in dogs affect perceived personality traits in dogs. Considering that wolves are large predators that potentially cause harm to people, early dogs may have evolved personality traits that are more acceptable to human society. Moreover, dogs may be more adaptable if they evolve infantile traits and elicit caregiving responses from humans. In this regard, a dog's friendliness and immaturity would be preferred by humans; this has also been suggested in recent findings on dog domestication. First, according to the emotional reactivity hypothesis, the selection for ‘friendliness’ or ‘tameness’ played a major role in the early stage of domestication in modern dogs [19,20]. This hypothesis is based on farm-fox experiments, which demonstrated that selective breeding of friendly foxes has not only a genetic basis for friendliness to humans, but also produces individuals with dog-like morphological traits, including white coats, floppy ears, and curly tails [21]. Second, dog evolution involves neoteny and paedomorphosis, which is characterized by the retention of juvenile traits at maturity. Previous studies have suggested that domestic dogs exhibit more juvenile morphological traits (e.g. shortened muzzles and smaller canines) and social behaviour (e.g. increased play and lower intensity of aggression) than wolves and that the variability of juvenile morphological traits among dog breeds covaries with their signalling behaviour [22–24]. Similarly, dog domestication may relate to the baby schema concept [25], in which a set of infantile traits (e.g. large, round-shaped eyes and forehead) have been shown to affect cuteness perception and elicit caretaking behaviour from parents [26]. Humans perceive images of dogs and cats with infantile facial features as cute, indicating that a preference for infantile facial configurations may emerge in other species [27]. Based on these findings, we assume that the human preference for friendly and immature dogs has contributed to dog evolution.

Relatedly, Nagasawa *et al*. [28] showed that mutual gaze between dogs and humans, but not between wolves and humans, triggers an oxytocin release in both species. This suggests that eye contact behaviour, which is regulated by an endocrine basis comparable to mother–infant bonding in humans (e.g. [29]), plays an important role in attachment between dogs and humans. The eye morphology underlying the gazing behaviour between dogs and humans may be associated with the domestication of dogs, but little attention has been paid to the changes in eye morphology between dogs and wolves. Many comparative studies on dogs and wolves have shown that, compared with wolves, dogs can use spontaneous eye contact behaviour toward humans in the context of the need for cooperative help from humans [19,30,31]. Moreover, a recent study showed that dogs have evolved their facial muscles, developing more expressive eyes than wolves [32]. These studies suggest that face-to-face communication with humans via direct gaze has been enhanced during dog domestication, which may be underlaid by facial morphological traits affecting human preference for dogs over wolves.

Here, we focus on the effect of domestication and hypothesize that dark-eyed dogs would be perceived as more friendly and immature by humans over light-eyed dogs, which resemble their wild ancestral species, wolves. Our hypothesis on the iris colour in dog eyes is based on the findings suggesting that the iris–pupil contrast (and especially pupil size) can change social signalling and perceived personality traits. Perea-García *et al*. [11] highlight that little attention has been paid to the variability of the iris and pupil in primates and list the potential sociocognitive functions. First, pupil size may reflect emotional and physiological states in humans: dilation of pupil size is associated with the perception of positive emotion (e.g. happiness), whereas constriction of the pupil is linked to the perception of negative emotion (e.g. anger, sadness) [33,34]. Second, pupil size may contribute to personality judgement and interpersonal trust in humans. For example, people with large pupils are commonly rated as more attractive and activate neural responses in the amygdala [35,36], and a recent study demonstrates that human participants evaluate those with dilating pupils as more friendly, attractive and trustworthy and behave more cooperatively with them in the trust game [37]. These studies suggest that the perceived larger pupil may elicit an empathetic response and helping behaviour of the observer. Given these results, we assume that (i) dark irises in dogs make it difficult to detect changes in pupil size (i.e. especially in terms of pupillary constriction) and thereby mask or attenuate threatening signalling (e.g. anger) to humans, and that (ii) humans overestimate pupil size in dogs with darker iris, which consequently leads them to assign more positive personality traits to those dogs.

Interestingly, eye coloration and its association with social signalling functions have been proposed in great apes. For example, Perea-García *et al*. [9] demonstrate that bonobos have darker iris (and lighter sclera), while chimpanzees have lighter iris (and darker sclera). In the context of this study, the external eye morphology of the chimpanzee is analogous to that of a wolf, whereas external eye morphology of the bonobo is analogous to that of a dog. Assuming that selection against aggression has contributed toward the evolution of bonobos and dogs [24], their similar eye morphology can be thought of as having evolved in parallel in both species. Overall, the lighter iris would increase arousal on an interactant upon eye contact, while the darker iris would send less threatening signals. Recently, Caspar *et al*. [38] tested the hypothesis that the depigmentation of the sclera has been induced by domestication, but there are no differences in scleral phenotypes between domesticated and their wild species. However, little attention has been paid to the domestication of iris colour, which is the focus of the current study.

Thus, the present study aimed to explore a possible wolf–dog difference in eye (iris–pupil contrast) colour using photo image analysis (Study 1) and test whether the human perception of dogs is affected by a dog's eye colour difference (Studies 2 and 3). More specifically, we expected that the eye colour of dogs would be darker than that of wolves and that dogs with darker irises (i.e. dog-like) would be perceived as more friendly and immature than dogs with lighter irises (i.e. wolf-like), which may be responsible for the human acceptance of dogs.

## 2. Material and methods

### 2.1. Study 1: comparative image analysis on the eye colour of wolves and dogs

#### 2.1.1. Samples

We analysed high-resolution facial images of 22 grey wolves and 81 domestic dogs. All images were obtained from websites such as ARKive and Wikimedia Commons. We selected image files in which the eye regions were visible and photographed in the outdoor environment under natural daylight for both species. To minimize regional and subspecies biases, we selected images of wolves from as many shooting areas as possible and included individuals with various coat colours, such as black and white. Similarly, we selected images of 35 major dog breeds to include as many different breeds with various iris colours, including yellow, as possible in the analysis.

#### 2.1.2. Image analysis

We analysed the iris colour difference between wolves and dogs based on the method applied by Ueda *et al*. [15]. We used the CIE LAB colour space in our image analysis since it simulated human trichromatic colour vision. In this colour system, the L dimension represents lightness (or greyscale) and the A and B dimensions represent red–green and blue–yellow colour components, respectively. We analysed one eye which was illuminated by bright light sources in each image. We traced the iris and pupil areas using the freehand selection tool in Adobe Photoshop. We selected each area as broadly as possible but did not include reflections of the light source in the eye. We then measured the mean values of L, A and B components in each selected area. Finally, we calculated contrast values between the iris and pupil region (the iris–pupil contrast) in each image for the L, A and B components, using the following formula: (values of pupil area − values of iris area) / (values of pupil area + values of iris area).

#### 2.1.3. Statistical analysis

To test species differences in eye colour, we performed Welch's t-tests with species as a between-subject factor and the iris–pupil contrast for L, A and B values as dependent variables.

### 2.2. Study 2: effect of dog's eye colour on human perception of dogs

#### 2.2.1. Image stimuli

**Figure 2.**
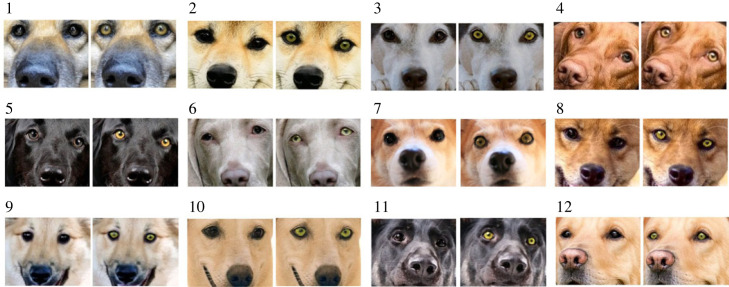
Thumbnails of image stimuli used in Study 2. A total of 24 dog facial images (12 dog images × 2 eye colours; ID 1–12) were used as image stimuli to be rated by human participants.

We created dogs’ facial images in which human participants rated their impressions. A total of 12 original images of dogs were obtained from royalty-free stock media websites (photoAC, Pixabay). We selected high-resolution images where both eyes of the dogs were clearly visible. To eliminate the effects of morphological traits other than eye colour, we used images of dogs with various breeds and coat colours and cropped the image to the region where both eyes and their muzzle were included. As illustrated in figure 2, original dog images of six pure breeds (two Labrador retrievers, Shiba, Vizsla, Weimaraner and Welsh corgi) and six mongrel dogs (or unidentified breeds) were used. Finally, we recoloured the eyes on 12 facial photographs of dogs from the original iris colour to black (dark-eyed) and yellow (light-eyed) using Adobe Photoshop. Thus, we created 24 dog facial images (12 dog images × 2 eye colours; ID 1–12) and used them as image stimuli to be rated by human participants.

#### 2.2.2. Questionnaire

We developed printed questionnaires to rate the human perception of dog images. The questionnaire consisted of image stimuli of dogs and rating scales. The rating scales were divided into two sections: personality traits of dogs in the image and attitudes of acceptance toward the dogs.

For personality trait ratings, we selected 10 pairs of items of the dog's personality traits representing the friendliness–aggressive dimension (nonaggressive–aggressive, uneasy–easygoing, unfriendly–friendly, unkind–kind, unsociable–sociable) and the immaturity–maturity dimension (unconfident–confident, dependent–independent, unintelligent–intelligent, immature–mature, untrustworthy–trustworthy). For attitudes toward the acceptance of dogs, we asked participants to rate the following two questions: ‘How much would you like to interact with the dog in the image?’ and ‘How much would you like to keep the dog in the image?’ Participants indicated the extent to which an item applied to each dog's image on a six-point scale (0–5) for personality ratings and on a four-point scale (0–3) for attitude ratings.

Finally, we developed four versions of the questionnaire (A, B, C and D) with six images of dogs included in each version. We included three images each of dark-eyed and light-eyed dogs in one version of the questionnaire but did not include images of the same dogs. We have provided one version of the questionnaire (translated into English) in the electronic supplementary material.

#### 2.2.3. Participants

We recruited the study participants via email and messenger applications through acquaintances of one of the authors (E.S.). A total of 76 participants consisting of 40 females and 34 males (two participants chose the option of ‘do not answer gender’) agreed to participate in the questionnaire survey. The participants were mainly students of the Teikyo University of Science, aged 18–38 years (average age of 21.742 years). To reduce the burden of rating, we asked the participants to fill out one of four versions of the questionnaire, therefore, each participant rated only six dog images.

#### 2.2.4. Data analysis

To estimate the structure of the personality trait ratings of dogs, we performed a factor analysis using data obtained from all participants (*N* = 456 [76 participants × 6 facial images]). To determine the number of factors extracted, we performed parallel and factor analyses using the maximum-likelihood method with varimax rotation. According to the results of the factor analysis (see Results), we calculated the average of the two scale scores (friendliness and maturity; see Results) of dog personality ratings for each of the 24 dog facial images.

To test the effect of eye colour on the personality ratings of dogs, we performed paired t-tests with eye colour difference (dark-eyed versus light-eyed) in the same dog facial images as an independent variable (within-subject factor) and two scale scores of friendliness and maturity as dependent variables. Additionally, we determined the effect of eye colour on each personality item using MANOVA tests.

Linear mixed models (LMMs) were used to determine the factors influencing the participants' acceptable attitudes toward dogs. We included eye colour (dark-eyed versus light-eyed) and scale scores for friendliness and maturity as explanatory variables (fixed factors), and the ID of dog images as the random factor. The response variables were the ratings of the questions ‘How much would you like to interact with the dog in the image?’ and ‘How much would you like to keep the dog in the image?’. We applied a Gaussian error structure when constructing the parameter estimates using LMMs. To test the fixed effect of each explanatory variable, we performed a likelihood ratio test with Wald Chi-square statistics (Type III test). Analyses were conducted using R v. 4.0.3 [39].

### 2.3. Study 3: replication of study 2

To assess the robustness of the findings obtained from Study 2, we conducted a replication study (Study 3) with the same general procedures, but with different questionnaire designs and participants. Study 2 used printed questionnaires to rate the human perception of dog images, whereas Study 3 used online questionnaires using Google Forms. We developed four versions of the online questionnaire using the same rating scales and order of items as in Study 2. For Study 3, we recruited 66 participants, 41 females and 25 males, aged 18–68 years (average age of 39.58 years). The participants did not overlap with those in Study 2 and included participants (*N* = 50, 30 females and 20 males, mean age = 45.93, range = 18–68) who were not university students. The participants responded to the questionnaire on their own devices (smartphones or PCs), and the size and resolution of the photo stimuli varied. We expected that differences in response formats and samples would not affect human perception and would thus yield results similar to that of Study 2. Data analysis was the same as that in Study 2.

## 3. Results

### 3.1. Study 1: comparative image analysis on the eye colour of wolves and dogs

**Figure 3.**
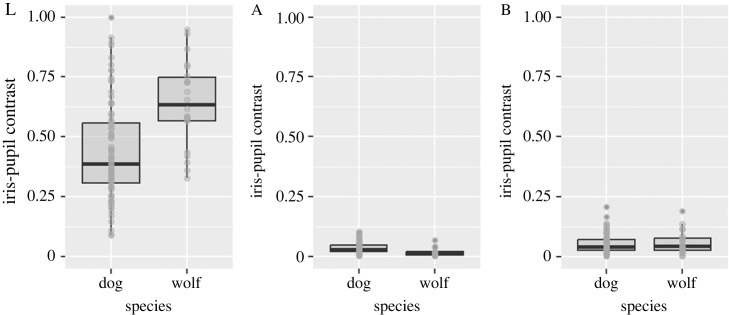
Iris colour difference between dogs and wolves by image analysis in Study 1. Box plots show dog–wolf differences in the iris–pupil contrast values for the luminance (L), A and B components.

Figure 3 shows the species differences in the iris–pupil contrast values for L, A and B components between wolves and dogs. The iris–pupil contrast of the L (*t* = 4.495, d.f. = 37.775, *p* < 0.001, 95%CI = 0.110–0.289, *d* = 0.993 [95%CI = 0.497–1.489]) and the *A* values (*t* = 4.911, d.f. = 35.730, *p* < 0.001, 95%CI = 0.015–0.037, *d* = 1.124 [95%CI = 0.623–1.626]) differed significantly between wolves and dogs. The contrast values of the B component did not differ significantly between species (*t* = 0.361, d.f. = 28.887, *p* = 0.721, 95%CI = −0.019 to 0.028, *d* = 0.098 [95%CI = −0.379 to 0.575]). These results indicate that the iris colour of dogs is darker and more reddish than that of wolves.

### 3.2. Study 2: effect of dog eye colour on human perception of dogs

Parallel analysis indicated a two-factor solution, which was consistent with a visual inspection of the scree plot. As shown in table 1 (left column), the factor analysis extracted two independent factors (factor 1 and factor 2) that accounted for 0.534 of the total variance. Items with higher factor 1 loading included kindness, sociability, easygoing, friendliness and non-aggressiveness, whereas those with higher factor 2 loadings included trustworthiness, confidence, independence, intelligence and maturity. Since the extracted structure corresponded to our hypothesis, we labelled factor 1 and factor 2 as ‘friendliness’ and ‘maturity,’ respectively.

**Table 1.** Factor structure and factor loadings for ten personality items in dog images. Factor loadings greater than 0.4 are shown in boldface type.

items	study 2			study 3		
	factor 1	factor 2	*h^2^*	factor 1	factor 2	*h^2^*
sociable	**0.826**	0.013	0.682	**0.843**	−0.054	0.713
kind	**0.822**	−0.105	0.686	**0.868**	−0.119	0.767
friendly	**0.780**	−0.040	0.610	**0.816**	−0.033	0.667
easygoing	**0.779**	−0.029	0.608	**0.843**	−0.188	0.745
aggressive	**−0.605**	0.161	0.393	**−0.703**	0.251	0.557
trustworthy	0.018	**0.798**	0.637	−0.024	**0.743**	0.552
confident	0.022	**0.684**	0.469	−0.064	**0.713**	0.513
independent	−0.275	**0.662**	0.514	−0.316	**0.687**	0.572
intelligent	0.030	**0.620**	0.386	0.012	**0.752**	0.565
mature	−0.321	**0.506**	0.359	−0.298	**0.573**	0.417
sum of squared loadings	3.120	2.224		3.523	2.542	

**Figure 4.**
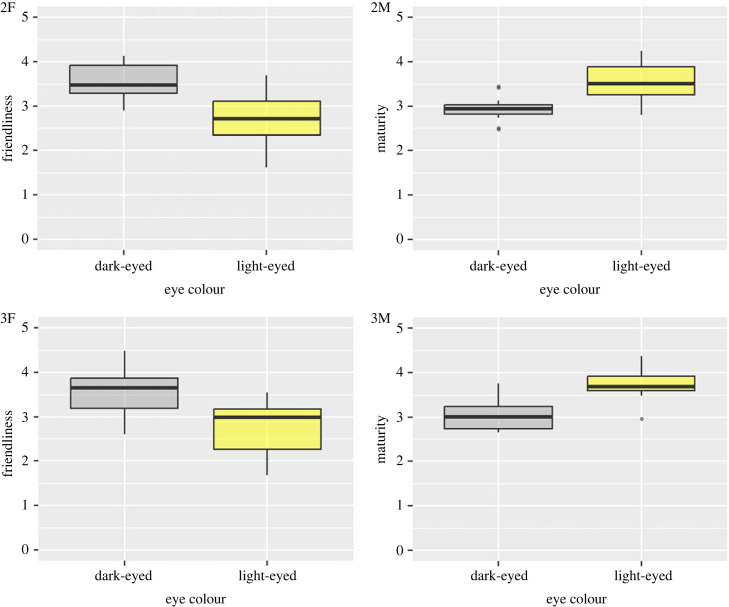
The effect of eye colours on the personality ratings of dogs. Box plots show the friendliness and maturity scores according to eye colour (dark-eyed versus light-eyed) in Study 2 (upper: 2F and 2M) and Study 3 (lower: 3F and 3M).

Figure 4 (2F and 2M) show the effects of eye colour on dog personality ratings in Study 2. We found that the facial images of dark-eyed dogs had higher friendliness scores than those of light-eyed dogs (*t* = 4.708, d.f. = 11, *p* < 0.001, 95%CI = 0.459–1.266, *d* = 1.7408 [95%CI = 0.747–2.735]). We also found that the facial images of dark-eyed dogs were rated lower in maturity scores than those of light-eyed dogs (*t* = −7.484, df = 11, *p* < 0.001, 95%CI = −0.797 to −0.435, *d* = −1.674 [95%CI −2.658 to −0.690]). The supplemental tables and figures (see electronic supplementary material) are the MANOVA results indicating that dark-eyed dogs were significantly rated more easygoing, friendly, kind, sociable, non-aggressive, unconfident, dependent, unintelligent, immature and untrustworthy than light-eyed dogs.

Table 2 (right column) shows the LMM results indicating the factors that influenced the participants' acceptable attitudes toward dogs. We found that a dog's eye colour had no significant effect on the attitudes of wanting to ‘interact’ with (*β* = 0.034, 95%CI = −0.159–0.225) and ‘keep’ the dog (*β* = 0.098, 95%CI = −0.109–0.303). For dog personality ratings, the friendliness scale scores were significantly associated with both the attitudes of wanting to ‘interact’ with (*β* = 0.304, 95%CI = 0.160–0.446) and ‘keep’ the dog (*β* = 0.340, 95%CI = 0.183–0.503). The maturity scale scores did not have a significant effect on both the attitudes of wanting to ‘interact’ with (*β* = −0.062, 95%CI = −0.277–0.151) or ‘keep’ the dog (*β* = 0.034, 95%CI = −0.228–0.277). These results showed that dogs with higher friendliness scores, but not maturity scores, received more accepting attitudes from human participants.

**Table 2.** The LMM results for the effect of dog eye colour and personality ratings on the attitudes of wanting to ‘interact’ with and wanting to ‘keep’ the dog images. Significant results (*p <* 0.05) are shown in bold.

samples	study 2			study 3		
explanatory variables	d.f.	Wald *χ*^2^	*p*	d.f.	Wald *χ*^2^	*p*
response variable: wanting to ‘interact’ with the dog						
eye colour	1	0.108	0.742	1	0.297	0.586
friendliness	1	**15.710**	**<0.001**	1	**21.044**	**<0.001**
maturity	1	0.299	0.585	1	2.134	0.144
response variable: wanting to ‘keep’ the dog						
eye colour	1	0.793	0.373	1	2.378	0.123
friendliness	1	**16.864**	**< 0.001**	1	**9.263**	**0.002**
maturity	1	0.069	0.794	1	**4.791**	**0.029**

### 3.3. Study 3: replication of study 2

As shown in table 1 (right column), the factor analysis extracted two factors (friendliness and maturity) that were similar to the factor structure in Study 2. As shown in figure 4 (3F and 3M), the facial images of dark-eyed dogs were rated significantly higher in friendliness scores (*t* = 6.751, d.f. = 11, *p* < 0.001, 95%CI = 0.561–1.104, *d* = 1.398 [95%CI = 0.453–2.342]) and significantly lower in maturity scores (*t* = −5.742, d.f. = 11, *p* < 0.001, 95%CI = −0.982 to −0.438, *d* = −1.944 [95%CI = −2.971 to −0.916]) than those of light-eyed dogs, which is consistent with the results of Study 2. The supplemental tables and figures (see electronic supplementary material) are the MANOVA results indicating that dark-eyed dogs were significantly rated more easygoing, friendly, kind, sociable, non-aggressive, unconfident, dependent, unintelligent, immature and untrustworthy than light-eyed dogs. Table 2 (right column) summarizes the LMM results, indicating the effects of dog eye colour and personality ratings on attitudes toward dog images. The dog's eye colour did not have a significant effect on the attitudes of wanting to ‘interact’ with (*β* = −0.052, 95%CI = −0.238–0.125) and ‘keep’ the dog (*β* = −0.189, 95%CI = −0.436–0.041). The friendliness scale scores were significantly associated with both of the attitudes of wanting to ‘interact’ with (*β* = 0.302, 95%CI = 0.179–0.435) and ‘keep’ the dog (*β* = 0.257, 95%CI = 0.099–0.429). The maturity scale scores did not have a significant effect on the attitudes of wanting to ‘interact’ with (*β* = 0.148, 95%CI = −0.040–0.337) but had a significant effect on the attitudes of wanting to ‘keep’ the dog (*β* = 0.284, 95%CI = 0.041–0.530). These results show that dog images with higher friendliness and lower maturity scores received more accepting attitudes from human participants.

## 4. Discussion

The present study explored the wolf–dog differences in iris colour and assessed whether the eye colour of dogs affects the human perception of personality in dogs and human attitudes toward the acceptance of dogs. We found that the irises of dogs were significantly darker than those of wolves (Study 1). Moreover, we found that the facial images of dark-eyed dogs were perceived as more friendly and immature than those of light-eyed dogs and that the friendliness of the dogs facilitated acceptable attitudes toward them (Study 2 and Study 3). These results support our hypothesis that dark eye colour in modern dogs may have been favoured by artificial selection during domestication from wolves to dogs and suggest that dogs with dark-coloured eyes may have evolutionarily adapted by acquiring a facial trait that sends a non-threatening gaze signal to humans as discussed below.

Our image analysis revealed that the iris colour of dogs was significantly darker and reddish than that of wolves. This result is the first empirical data supporting the qualitative documentation that the ‘dark-brown’ eyes of dogs differ from the ‘yellow’ eyes of wolves [18]. In addition, these data are consistent with the breed standards provided by the Kennel Club and the American Kennel Club that recommend darker eye colours over lighter ones in most pure-bred dogs. Compared with the results of studies in primates, this result may be congruent to the findings of Perea-García *et al*. [9], which showed that the iris of the bonobo is darker than that of the chimpanzee. The self-domestication hypothesis argues that selection against aggression may contribute to behavioural and phenotypic traits commonly expressed in humans, bonobos and dogs [24]. Iris colour pigmentation may be one candidate for morphological traits associated with the evolutionary history at least in these self-domesticated species. However, the neural-crest cell hypothesis of domestication assumes that a process of domestication leads to depigmentation rather than an intensification of pigmentation [40,41]. Moreover, a recent study did not support the correlation between depigmentation of the sclera and domestication events [38]. Thus, this issue should be addressed via more comprehensive future studies.

In terms of the evolutionary drive for dogs' darker eyes, we argue in favour of the domestication hypothesis, which assumes that human preferences may have shaped the evolution of darker eyes in dogs. That is, humans prefer dark-eyed dogs over light-eyed dogs. Consistent with this assumption, our questionnaire survey revealed that human participants perceived facial images of dark-eyed dogs as more friendly and more immature than those of light-eyed dogs. Moreover, facial images of dogs perceived as more friendly were shown to enhance acceptable attitudes (the degree of wanting to interact with and keep the dog) toward dogs. These results were replicated in the independent analyses (Studies 2 and 3) using different participants, indicating that human perception of dog eye colour is not substantially influenced by variability in the attributes of participants. Thus, the findings of our study show that a dog's dark eye enhances the human perception of friendliness and immaturity.

The association between perceived friendliness and dark eyes in dog images is compatible with our assumption that dark irises minimize the detectability of changes in pupil size, attenuating negative emotional signalling to humans: overall, this cascade of events culminates in an overestimation of pupil size, which results in the perception of positive personality traits. This possibility stems from the fact that humans have a cognitive tendency to perceive emotional arousal based on iris–pupil contrast and make inferences about a person's personality [33–37]. Recent experimental studies manipulating the sclera colour in hominoid or human faces show that white sclera positively correlated with attractiveness, trustworthiness and low aggressiveness [42] and that the preference for white sclera emerges in early developmental age [43]. Few studies have focused solely on the effect of iris colour on the perception of a person's personality, but there is evidence that facial morphological features that typically accompany brown eyes lead to greater perceived trustworthiness [44]. Our study provides the first evidence showing the influence of the iris colour pattern on the perception of cooperative personality, suggesting that personality judgement based on the iris–pupil coloration of subjects extended to other species of domesticated companions (i.e. dogs).

We found that darker eyes in dogs are linked not only to the perception of friendliness but also to immaturity. Two independent factors (i.e. friendliness and maturity) extracted in this study suggest that personality assessment of dog facial images by humans is based on not a general factor of positive impression toward the dog but on the traits both of lower emotional reactivity and paedomorphism, traits potentially tied to the evolution of dogs in the context of domestication (e.g. [24]). Particularly, our study shows that darker iris colour is associated with immaturity including items of untrustworthy, unconfident, dependent, unintelligent and immature. This suggests that dogs with darker irises are regarded as weak and to-be-protected beings rather than as cooperative partners, which potentially elicits caring responses from the observer. Although the proximate mechanism underlying the result is unclear, darker eye colour may convey ‘child-like’ signals to caregivers. One possibility comes from that humans may estimate a person's age based on pupil diameter since the pupil size (both the baseline and light-adapted pupil size) in humans decreases with age [45,46]. Given that dogs' darker and lighter eyes (i.e. iris and pupil) appear to have dilated pupils, whereas dogs’ lighter eyes appear to have constricted pupils, this may lead to dogs with darker eyes being regarded as younger compared with those with lighter eyes. Similarly, more of the white sclera is visible in human adults than in infants [2], which may relate to our results. The lighter iris and pupil of a wolf's eye resembles the white sclera and iris–pupil of humans, respectively: specifically, wolves appear to have a smaller pupil surrounded a widely exposed and light sclera. By contrast, the dogs' eyes appear to have a single dark iris and pupil, giving the illusion of a larger dark eye with no sclera. Hence, the dog-like eyes may be estimated to be younger than the wolf-like ones.

However, although dogs’ eye colour was significantly correlated with dog personality judgements, no significant association was found between dogs' eye colour and acceptance attitudes toward dogs. This suggests that a dog's darker eye colour is not a critical trait that directly increases human acceptance. In practice, a dog's friendliness and immaturity can be estimated based on its whole-body morphological traits (e.g. body size and proportions, head and muzzle length, facial structure, leg length, ear, tail and hair morphology), the eye colour being only one of these traits. Although not fully examined in this study, it is likely that the decision to adopt a dog is determined by multiple traits. Furthermore, it has been shown that, unlike facial photographs of human babies, those of dogs and cats elicit cuteness perception from humans but do not highly activate the brain's reward system [47,48]. A study on cats in an animal shelter also showed that the degree of the baby schema of individual cats did not significantly influence the length of stay in the shelter [49]. These findings suggest that although the human perception associated with cuteness generalizes to other animal species, it may not fully evoke motivation for actual nurturing behaviour in humans when compared with its own species (the human baby).

We should note the alternative explanation for our result of the perception of positive personality traits from dogs with darker eyes: the familiarity effect. This effect may work in two directions (i) that humans prefer dark-eyed dogs that are more frequently observed, and (ii) that humans prefer dogs’ eye colours similar to their own. The former case has been commonly pointed out as an unresolved issue in those studies using subjective ratings by human subjects [42–44], which should be investigated by controlling the stimulus familiarity in subsequent studies. The current study used various photographs of several dog breeds (i.e. light-eyed breeds, dark-eyed breeds and mongrels) as the original materials, which at least minimized the degree of the unusual combination of iris colour and facial configuration in dog images. For the latter, all participants of this study were conducted with Japanese people whose iris was dark brown or almost black. This might affect the preference for dark-eyed dogs. A future cross-cultural study with participants with variable iris colour patterns and ethnicity allows us to test whether the ‘ethnocentrism’ preference for dog eyes is present or not. Nevertheless, the present study did not evaluate a single ‘social-desirability’ factor for dog personality, but rather two independent factors of the friendliness–aggressive dimension and the immaturity–maturity dimension. Particularly, darker eyes in dogs are positively correlated with friendliness, while negatively correlated with the maturity factor (confident, independent, intelligent, mature, reliable), which is considered desirable in human personality traits. Thus, we assume the familiarity effect is partially limited.

There are other limitations to our study. First, the samples of dog facial images are limited in that we prioritized photographic clarity and used the facial images of only 33 major pure-bred dog breeds available online. There are more than several hundred recognized pure breeds of modern dogs, as well as many mongrel dogs with complex pedigrees and free-ranging dogs worldwide. In particular, a small number of breeds (e.g. Border collie, Shetland sheepdog and Siberian husky) have lighter iris colours, including yellow, blue and amber, which seem to be related to variations in coat colour. Wolves also have a wide range of habitats and high morphological diversity. Therefore, it may be necessary to collect images that fully capture the phenotypic diversity of both wolves and dogs, to allow for more rigorous comparisons. Nevertheless, the approach used in this study is a reasonable first step to determine the overall trends in eye colour (i.e. lightness) of representative pure-breds, and we believe that differences in iris colour between dogs and wolves would be reproduced in other samples. Indeed, our sample included several ancient-type breeds (e.g. Afghan hound, Akita, and Alaskan malamute), a breed group genetically close to wolves, and even those with dark-coloured eyes, which are recommended in their breed standards. This also supports the idea that the darker eyes of dogs may have emerged at an early stage of domestication, diverging from wolves.

Second, refinement of the analyses and consideration of other approaches are important for future research. For example, in the context of primate eye morphology research, early discoveries of human eye uniqueness by Kobayashi and Kohshima [1] have been updated by subsequent studies applying new image analysis techniques (e.g. [8]) and anatomical examinations [2]. For instance, experimental studies that unify the photographing conditions of eye images, and anatomical studies that directly observe the morphological structure of dog eyes, may be necessary. Future studies on the human perception of dogs should use not only subjective assessments using questionnaires but also behavioural indicators. Previous studies on non-human primates have shown that subjects prefer infantile features over adult features in terms of gaze duration, approach frequency and reaction time [27,50–52], and these methodologies could be applied to the study of dogs and other species.

Finally, we should consider the alternative functional mechanisms underlying iris pigmentation in dogs. Note that Perea-García *et al*. [10] demonstrate that the eye morphology in primates is associated with the ecological pressures they are subjected to (i.e. terrestriality, latitude), pointing to the importance of photoprotective and circadian functions on external eye coloration in primates. Similarly, environmental or geographical factors, such as adaptation to ultraviolet radiation, may have been involved in melanin pigment synthesis in dogs and wolves, resulting in the emergence of a darker iris. Wolves inhabit the Northern Hemisphere of Eurasia and North America, whereas domestic dogs expanded to all continents, except Antarctica [17]. Hence, it is possible that the ability to synthesize melanin for protection against ultraviolet radiation was selected during the process of southward migration, which may have contributed to the acquisition of darker eyes in dogs. This case is largely consistent with the assumption of this study that darker eyes evolved during domestication. Indeed, iris colour is a single trait with almost no intraspecific diversity in wild species (but see [6]). However, iris colour exhibits high levels of heterogeneity in humans and the species they have domesticated, suggesting that iris colour variation is largely driven by domestication and self-domestication [53].

In conclusion, our results suggest that the iris colour of dogs is darker than that of wolves, and that dark eyes of dogs positively affect human perception toward dogs. We can assume that darker eye appearance in modern dogs is associated with the following evolutionary scenarios: (i) wolves have evolved an external eye appearance (i.e. lighter iris) that is adaptive under natural selection (e.g. conspecific communication, photoprotection, or a combination of these), (ii) as ancestral dogs lived with humans, their natural selection pressures have been relaxed, (iii) artificial selection by human preference has shaped some traits in dogs, and (iv) modern dogs have evolved an external eye appearance (i.e. darker iris) that allows them to be perceived as friendly and immature by humans. Overall, dogs with dark eyes may have evolved the trait largely as means to send non-threatening gaze signal to humans.

## Data Availability

Supplementary material is available online [54].
